# 2-(5-Fluoro-2,3-dioxoindolin-1-yl)ethyl 4-methyl­piperazine-1-carbodithio­ate

**DOI:** 10.1107/S1600536811052494

**Published:** 2011-12-10

**Authors:** Yao Wang, Hui-Hui Lin, Sheng-Li Cao

**Affiliations:** a Department of Chemistry, Capital Normal University, Beijing 100048, People’s Republic of China

## Abstract

In the title compound, C_16_H_18_FN_3_O_2_S_2_, the methyl­piperazine ring adopts a chair conformation, while the (2,3-dioxoindolin-1-yl)ethyl unit is linked to one of the N atoms of the piperazine ring *via* the carbodithio­ate group. In the crystal, each mol­ecule is linked to its neighbors within the (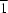
03) plane through weak C—H(methyl­ene)⋯O, C—H(ar­yl)⋯O and C—H(methyl­ene)⋯S inter­actions. Perpendicular to this plane mol­ecules are connected through inter­molecular short N⋯π(pyrrole ring) contacts [N⋯C centroid = 3.232 (2) Å], another set of C—H(methyl­ene)⋯O inter­actions and through short contacts between carbodithio­ate S atoms and the pyrrole rings [C⋯centroid = 3.695 (3), S⋯centroid = 3.403 (2) Å].

## Related literature

For background to indoline-2,3-dione and its derivatives, see: Bhattacharya & Chakrabarti (1998 ▶); Sridhar & Ramesh (2001 ▶); Medvedev *et al.* (1996 ▶) and to dithio­carbamates, see: Ozkirimli *et al.* (2005 ▶); Cao *et al.* (2005 ▶); Gaspari *et al.* (2006 ▶). For analogues of 5-fluoro­indoline-2,3-dione, see: Wang *et al.* (2010 ▶). For N⋯π contacts, see: Black *et al.* (2007 ▶). For van der Waals radii, see Bondi (1964 ▶). For the thickness of phenyl rings, see: Malone *et al.* (1997 ▶). For C=O⋯π (pyrid­yl) contacts, see: Wan *et al.* (2008 ▶)
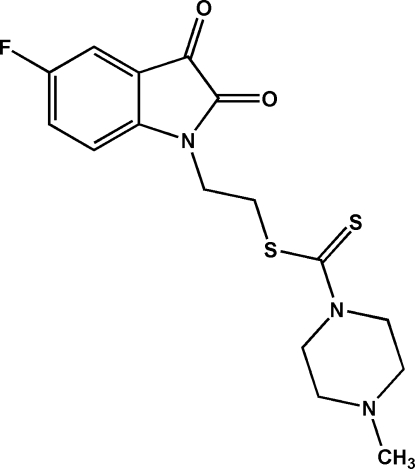


## Experimental

### 

#### Crystal data

C_16_H_18_FN_3_O_2_S_2_
*M* *_r_* = 367.45
Monoclinic, 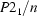

*a* = 10.0258 (4) Å
*b* = 15.9925 (6) Å
*c* = 11.0016 (5) Å
β = 106.656 (3)°
*V* = 1689.96 (12) Å^3^
*Z* = 4
Mo *K*α radiation
μ = 0.34 mm^−1^
*T* = 296 K
0.30 × 0.30 × 0.20 mm

#### Data collection

Bruker SMART APEXII CCD area-detector diffractometer
Absorption correction: multi-scan (*SADABS*; Bruker 2007 ▶) *T*_min_ = 0.658, *T*_max_ = 0.746
18809 measured reflections
3861 independent reflections
3058 reflections with *I* > 2σ(*I*)
*R*_int_ = 0.031

#### Refinement

*R*[*F* ^2^ > 2σ(*F* ^2^)] = 0.035
*wR*(*F* ^2^) = 0.100
*S* = 1.04
3861 reflections
217 parameters
H-atom parameters constrained
Δρ_max_ = 0.22 e Å^−3^
Δρ_min_ = −0.20 e Å^−3^

### 

Data collection: *APEX2* (Bruker, 2007 ▶); cell refinement: *APEX2* and *SAINT* (Bruker, 2007 ▶); data reduction: *SAINT*; program(s) used to solve structure: *SHELXS97* (Sheldrick, 2008 ▶); program(s) used to refine structure: *SHELXL97* (Sheldrick, 2008 ▶); molecular graphics: *SHELXTL* (Sheldrick, 2008 ▶); software used to prepare material for publication: *SHELXTL* and *PLATON* (Spek, 2009 ▶).

## Supplementary Material

Crystal structure: contains datablock(s) I, global. DOI: 10.1107/S1600536811052494/zl2432sup1.cif

Structure factors: contains datablock(s) I. DOI: 10.1107/S1600536811052494/zl2432Isup2.hkl

Supplementary material file. DOI: 10.1107/S1600536811052494/zl2432Isup3.cml

Additional supplementary materials:  crystallographic information; 3D view; checkCIF report

## Figures and Tables

**Table 1:** Hydrogen-bond geometry (Å, °)

*D*—H⋯*A*	*D*—H	H⋯*A*	*D*⋯*A*	*D*—H⋯*A*
C13—H13*B*⋯O2^i^	0.97	2.50	3.225 (2)	131
C12—H12*A*⋯O2^ii^	0.97	2.61	3.385 (2)	137
C15—H15*B*⋯O2^ii^	0.97	2.62	3.383 (2)	136
C1—H1*A*⋯O1^iii^	0.93	2.70	3.282 (3)	121
C2—H2*A*⋯O1^iii^	0.93	2.67	3.275 (2)	124
C12—H12*B*⋯S2^i^	0.97	2.97	3.866 (3)	155

Symmetry codes: (i) 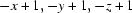; (ii) 
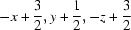
; (iii) 
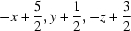
.

**Fig. 1.**
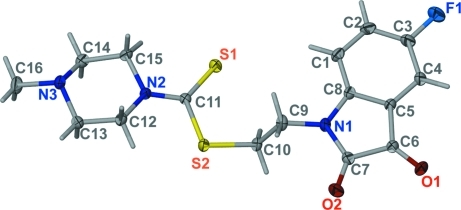
The title molecule with the atomic numbering scheme. The displacement ellipsoids of the non-hydrogen atoms are shown at the 30% probability level.

**Fig. 2.**
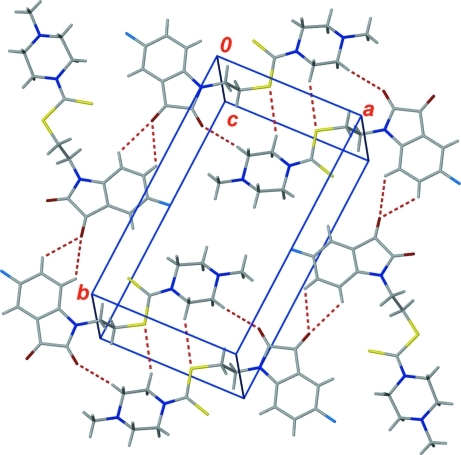
The intermolecular C—H(aryl)···O, C—H(methylene)···S and C—H(methylene)···O interactions of the title compound within the (-1 0 3) Miller plane. View perpendicular to this plane.

**Fig. 3.**
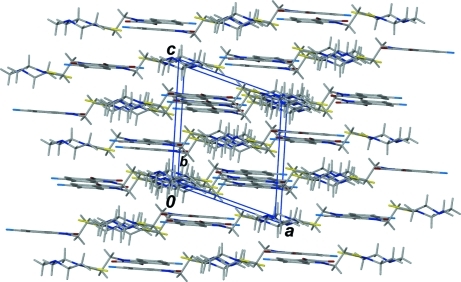
View down the b direction of the stacking structure of the title compound. All weak non-covalent interactions are omitted for clarity.

## supplementary crystallographic information

### Comment

Indoline-2,3-dione and its derivatives are well known for their broad spectrum biological and pharmacological properties including anticonvulsant (Bhattacharya & Chakrabarti, 1998), anti-inflammatory (Sridhar *et al.*, 2001) and anxiogenic (Medvedev *et al.*, 1996) activities. On the other hand, dithiocarbamates also exhibit a large range of biological activities such as fungicidal (Ozkirimli *et al.*, 2005) and antitumor activities (Cao *et al.*, 2005; Gaspari *et al.*, 2006). In an attempt to obtain compounds that might also exhibit antitumor properties, but possibly with increased potency and selectivity, we designed and synthesized the title compoud (C_16_H_18_N_3_O_2_FS_2_), which consists of an indole core with a dithiocarbamate side chain (Scheme 1). In the present context, we report the crystal structure of the new compound.

In the crystalline structure of the title compound, the 1-methylpiperazine ring adopts a chair conformation, while the indoline-2,3-dione ethyl moiety is linked to one of the N atoms of the piperazine ring via the carbodithioate group, with the ethyl group in a trans-conformation (N1—C9—C10—S2 torsion angle of 175.74 (11)°, Fig. 1). This trans-conformation differentiates the title compound from the related compound 2-(2,3-dioxoindolin-1-yl)ethyl-4-(4-nitrophenyl)piperazine-1-carbodithioate reported by us recently (Wang *et al.*, 2010), which was found to have a *gauge*-conformation with an N4(pyrrole)—C19—C20—S2 torsion angle of 66.16 (15)°. Through weak C13—H13B(methylene)···O2^i^, C—H(aryl)···O1^ii^ and C12—H12B(methylene)···S2^iii^ interactions each molecule is linked to its neighbors within the (-1 0 3) Miller plane (Table 1 and Fig. 2). Perpendicular to  this plane molecules are connected through intermolecular short N···π (pyrrole ring) contacts, another set of C—H(methylene)···O interactions (Table 1) and through short contacts between carbodithioate sulfur atoms and the pyrrole rings (C11═S1···Cg1^iv^, Table 2). A short contact is observed between the nitrogen atom N1 and the π-electron desnity of the pyrrole ring, with an N3···Cg^v^ (Cg = C5-C6-C7-N1-C8, (v) = -x+1.5, y+0.5, -z+1.5) distance equal to 3.232 (2) Å, which is shorter than the van der Waals distance (3.40 Å) on the basis of Pauling's value for the half thickness of phenyl rings (1.85 Å) (Malone *et al.*,1997) and the van der Waals radius of N (1.55 Å) (Bondi, 1964). It is comparable to the N(pyrazinyl)···centroid(pyrazinyl) distance of 3.05 Å in {[Ni(L)(NO_3_)_2_]}_∞_ (L = bis(2-pyraylmethyl)sulfide) reported by Black *et al.* (2007). Regarding the C11═S1···π contact (Table 2), the C═S bond is almost parallel to the pyrrole ring with a C11═S1···Cg1(pyrrole) angle equal to 86.42 (2)°, a contact mode similar to that of the C═O···π (pyridyl) contact in Cu(L)_2_(BF_4_)_2_ (L = 2,6-pyridinediylbis(3-pyridinyl)methanone) reported by Wan *et al.* (2008).

### Experimental

A suspension of 1-methylpiperazine (2.4 mmol), carbon disulfide (0.72 mL, 12 mmol) and anhydrous potassium phosphate (0.51 g, 2.4 mmol) in *N,N*-dimethylformamide (15 mL) was stirred at room temperature for 30 minutes. Then, 1-(2-bromoethyl)-5-fluoroindoline-2,3-dione (2 mmol) was added and stirring was continued for 3.5 h. The reaction mixture was poured into water (100 mL) and the resulting precipitate was separated by filtration and further purified by column chromatography on silica gel with dichloromethane/methanol = 95:5 (v/v) as the eluent to give the title compound (R_f_ = 0.44, m.p. 472.2-473.2 K; yield 78%). After two weeks, the orange crystals of the title compound were deposited by slow evaporation from a solution of dichloromethane/*N,N*-dimethylformamide 1:1 (v/v) at room temperature.

### Refinement

All H atoms were discernible in the difference electron density maps. Nevertheless, the hydrogen atoms were placed into idealized positions and allowed to ride on their respective carrier atoms, with C—H = 0.93 and 0.97 Å for aryl and methylene hydrogens, respectively. *U*_iso_(H) = 1.2*U*_eq_(C)_aryl_/_methylene_.

### Crystal data

**Table:**

C_16_H_18_FN_3_O_2_S_2_	*F*(000) = 768
*M**_r_* = 367.45	*D*_x_ = 1.444 Mg m^−^^3^
Monoclinic, *P*2_1_/*n*	Mo *K*α radiation, λ = 0.71073 Å
Hall symbol: -P 2yn	Cell parameters from 222 reflections
*a* = 10.0258 (4) Å	θ = 2.3–27.6°
*b* = 15.9925 (6) Å	µ = 0.34 mm^−^^1^
*c* = 11.0016 (5) Å	*T* = 296 K
β = 106.656 (3)°	Block, colorless
*V* = 1689.96 (12) Å^3^	0.30 × 0.30 × 0.20 mm
*Z* = 4	

### Data collection

**Table:**

Bruker SMART APEXII CCD area-detector diffractometer	3861 independent reflections
Radiation source: fine-focus sealed tube	3058 reflections with *I* > 2σ(*I*)
graphite	*R*_int_ = 0.031
CCD area detector scans	θ_max_ = 27.6°, θ_min_ = 2.3°
Absorption correction: multi-scan (*SADABS*; Bruker 2007)	*h* = −13→13
*T*_min_ = 0.658, *T*_max_ = 0.746	*k* = −20→20
18809 measured reflections	*l* = −11→14

### Refinement

**Table:**

Refinement on *F*^2^	Primary atom site location: structure-invariant direct methods
Least-squares matrix: full	Secondary atom site location: difference Fourier map
*R*[*F*^2^ > 2σ(*F*^2^)] = 0.035	Hydrogen site location: inferred from neighbouring sites
*wR*(*F*^2^) = 0.100	H-atom parameters constrained
*S* = 1.04	*w* = 1/[σ^2^(*F*_o_^2^) + (0.0506*P*)^2^ + 0.3797*P*] *P* = (*F*_o_^2^ + 2*F*_c_^2^)/3
3861 reflections	(Δ/σ)_max_ = 0.001
217 parameters	Δρ_max_ = 0.22 e Å^−^^3^
0 restraints	Δρ_min_ = −0.20 e Å^−^^3^

### Special details

**Table:**

Geometry. All esds (except the esd in the dihedral angle between two l.s. planes) are estimated using the full covariance matrix. The cell esds are taken into account individually in the estimation of esds in distances, angles and torsion angles; correlations between esds in cell parameters are only used when they are defined by crystal symmetry. An approximate (isotropic) treatment of cell esds is used for estimating esds involving l.s. planes.
Refinement. Refinement of F^2^ against ALL reflections. The weighted R-factor wR and goodness of fit S are based on F^2^, conventional R-factors R are based on F, with F set to zero for negative F^2^. The threshold expression of F^2^ > 2sigma(F^2^) is used only for calculating R-factors(gt) etc. and is not relevant to the choice of reflections for refinement. R-factors based on F^2^ are statistically about twice as large as those based on F, and R- factors based on ALL data will be even larger.

### Fractional atomic coordinates and isotropic or equivalent isotropic displacement parameters (Å^2^)

**Table:**

	*x*	*y*	*z*	*U*_iso_*/*U*_eq_	
S1	0.91389 (4)	0.66656 (2)	0.57656 (4)	0.04717 (13)	
S2	0.69048 (4)	0.53592 (2)	0.54985 (5)	0.04795 (14)	
N1	1.04768 (13)	0.40980 (8)	0.72003 (13)	0.0436 (3)	
N2	0.64449 (12)	0.69676 (7)	0.54089 (14)	0.0423 (3)	
N3	0.42553 (14)	0.81754 (8)	0.50085 (14)	0.0466 (3)	
O1	1.22167 (14)	0.21917 (7)	0.75006 (15)	0.0677 (4)	
O2	0.93355 (12)	0.28341 (8)	0.68042 (13)	0.0580 (3)	
F1	1.60973 (12)	0.46870 (8)	0.86069 (15)	0.0840 (4)	
C1	1.24078 (19)	0.51646 (9)	0.77343 (17)	0.0477 (4)	
H1A	1.1821	0.5626	0.7618	0.057*	
C2	1.3845 (2)	0.52580 (10)	0.80939 (19)	0.0554 (4)	
H2A	1.4233	0.5790	0.8224	0.066*	
C3	1.46982 (18)	0.45705 (11)	0.82584 (19)	0.0555 (5)	
C4	1.41990 (17)	0.37630 (10)	0.80778 (18)	0.0502 (4)	
H4A	1.4793	0.3305	0.8192	0.060*	
C5	1.27711 (16)	0.36716 (9)	0.77186 (16)	0.0419 (4)	
C6	1.19005 (16)	0.29208 (9)	0.74546 (17)	0.0465 (4)	
C7	1.03847 (16)	0.32457 (10)	0.70982 (17)	0.0449 (4)	
C8	1.18809 (15)	0.43610 (9)	0.75556 (15)	0.0397 (3)	
C9	0.92604 (17)	0.46265 (11)	0.70586 (17)	0.0488 (4)	
H9A	0.9553	0.5161	0.7465	0.059*	
H9B	0.8643	0.4365	0.7483	0.059*	
C10	0.84770 (16)	0.47709 (10)	0.56780 (17)	0.0446 (4)	
H10A	0.8247	0.4235	0.5257	0.054*	
H10B	0.9073	0.5069	0.5269	0.054*	
C11	0.74743 (15)	0.64129 (9)	0.55535 (15)	0.0368 (3)	
C12	0.50387 (16)	0.67350 (10)	0.5460 (2)	0.0531 (5)	
H12A	0.5005	0.6763	0.6331	0.064*	
H12B	0.4842	0.6164	0.5168	0.064*	
C13	0.39430 (17)	0.73083 (11)	0.4648 (2)	0.0545 (4)	
H13A	0.3903	0.7235	0.3763	0.065*	
H13B	0.3040	0.7163	0.4745	0.065*	
C14	0.55667 (18)	0.83880 (10)	0.47625 (18)	0.0507 (4)	
H14A	0.5765	0.8976	0.4946	0.061*	
H14B	0.5482	0.8297	0.3872	0.061*	
C15	0.67565 (16)	0.78703 (9)	0.55582 (18)	0.0470 (4)	
H15A	0.7590	0.7989	0.5309	0.056*	
H15B	0.6935	0.8024	0.6443	0.056*	
C16	0.3137 (2)	0.87270 (12)	0.4299 (2)	0.0615 (5)	
H16A	0.2276	0.8563	0.4449	0.092*	
H16B	0.3051	0.8686	0.3410	0.092*	
H16C	0.3350	0.9294	0.4575	0.092*	

### Atomic displacement parameters (Å^2^)

**Table:**

	*U*^11^	*U*^22^	*U*^33^	*U*^12^	*U*^13^	*U*^23^
S1	0.03141 (19)	0.0426 (2)	0.0657 (3)	−0.00238 (15)	0.01100 (18)	−0.00061 (18)
S2	0.03104 (19)	0.03047 (19)	0.0770 (3)	0.00221 (13)	0.00697 (18)	0.00188 (17)
N1	0.0375 (6)	0.0350 (6)	0.0536 (9)	0.0071 (5)	0.0055 (6)	0.0042 (6)
N2	0.0310 (6)	0.0303 (6)	0.0618 (9)	0.0001 (5)	0.0072 (6)	−0.0043 (6)
N3	0.0431 (7)	0.0370 (7)	0.0530 (9)	0.0105 (5)	0.0030 (6)	−0.0005 (6)
O1	0.0549 (7)	0.0274 (6)	0.1107 (12)	0.0032 (5)	0.0075 (7)	−0.0003 (6)
O2	0.0407 (6)	0.0551 (7)	0.0713 (9)	−0.0104 (5)	0.0047 (6)	0.0008 (6)
F1	0.0452 (6)	0.0693 (8)	0.1238 (12)	−0.0187 (5)	0.0025 (7)	0.0018 (7)
C1	0.0571 (9)	0.0290 (7)	0.0549 (10)	0.0053 (7)	0.0127 (8)	0.0033 (7)
C2	0.0618 (11)	0.0337 (8)	0.0655 (12)	−0.0110 (7)	0.0102 (9)	−0.0015 (7)
C3	0.0416 (9)	0.0483 (9)	0.0680 (12)	−0.0110 (7)	0.0019 (8)	0.0006 (8)
C4	0.0386 (8)	0.0371 (8)	0.0670 (12)	0.0029 (6)	0.0026 (8)	0.0035 (7)
C5	0.0388 (8)	0.0278 (7)	0.0531 (10)	0.0014 (6)	0.0039 (7)	0.0020 (6)
C6	0.0402 (8)	0.0306 (7)	0.0621 (11)	0.0000 (6)	0.0041 (7)	0.0013 (7)
C7	0.0378 (8)	0.0399 (8)	0.0516 (10)	−0.0002 (6)	0.0042 (7)	0.0028 (7)
C8	0.0403 (7)	0.0315 (7)	0.0434 (9)	0.0046 (6)	0.0058 (7)	0.0038 (6)
C9	0.0428 (8)	0.0481 (9)	0.0556 (11)	0.0139 (7)	0.0144 (8)	0.0060 (7)
C10	0.0357 (7)	0.0366 (7)	0.0582 (10)	0.0080 (6)	0.0083 (7)	−0.0004 (7)
C11	0.0326 (7)	0.0332 (7)	0.0411 (8)	0.0000 (5)	0.0047 (6)	−0.0016 (6)
C12	0.0321 (7)	0.0320 (7)	0.0921 (14)	0.0004 (6)	0.0130 (8)	−0.0041 (8)
C13	0.0341 (8)	0.0466 (9)	0.0742 (12)	0.0037 (7)	0.0014 (8)	−0.0134 (8)
C14	0.0519 (9)	0.0373 (8)	0.0588 (11)	0.0038 (7)	0.0092 (8)	0.0006 (7)
C15	0.0410 (8)	0.0300 (7)	0.0660 (11)	−0.0022 (6)	0.0090 (8)	−0.0053 (7)
C16	0.0582 (11)	0.0553 (11)	0.0632 (12)	0.0224 (9)	0.0050 (9)	0.0085 (9)

### Geometric parameters (Å, °)

**Table:**

S1—C11	1.6673 (15)	C4—H4A	0.9300
S2—C11	1.7747 (15)	C5—C8	1.397 (2)
S2—C10	1.7973 (15)	C5—C6	1.464 (2)
N1—C7	1.369 (2)	C6—C7	1.547 (2)
N1—C8	1.413 (2)	C9—C10	1.514 (2)
N1—C9	1.4550 (19)	C9—H9A	0.9700
N2—C11	1.3353 (19)	C9—H9B	0.9700
N2—C12	1.4746 (19)	C10—H10A	0.9700
N2—C15	1.4763 (19)	C10—H10B	0.9700
N3—C13	1.452 (2)	C12—C13	1.511 (2)
N3—C14	1.457 (2)	C12—H12A	0.9700
N3—C16	1.464 (2)	C12—H12B	0.9700
O1—C6	1.2056 (18)	C13—H13A	0.9700
O2—C7	1.2039 (19)	C13—H13B	0.9700
F1—C3	1.357 (2)	C14—C15	1.509 (2)
C1—C8	1.382 (2)	C14—H14A	0.9700
C1—C2	1.389 (3)	C14—H14B	0.9700
C1—H1A	0.9300	C15—H15A	0.9700
C2—C3	1.373 (3)	C15—H15B	0.9700
C2—H2A	0.9300	C16—H16A	0.9600
C3—C4	1.379 (2)	C16—H16B	0.9600
C4—C5	1.379 (2)	C16—H16C	0.9600
			
C11—S2—C10	103.29 (7)	C9—C10—S2	112.11 (11)
C7—N1—C8	110.95 (12)	C9—C10—H10A	109.2
C7—N1—C9	122.35 (14)	S2—C10—H10A	109.2
C8—N1—C9	126.50 (13)	C9—C10—H10B	109.2
C11—N2—C12	122.86 (13)	S2—C10—H10B	109.2
C11—N2—C15	120.31 (13)	H10A—C10—H10B	107.9
C12—N2—C15	114.61 (12)	N2—C11—S1	124.34 (11)
C13—N3—C14	107.92 (13)	N2—C11—S2	113.37 (11)
C13—N3—C16	110.97 (14)	S1—C11—S2	122.29 (8)
C14—N3—C16	110.77 (14)	N2—C12—C13	111.44 (14)
C8—C1—C2	117.62 (14)	N2—C12—H12A	109.3
C8—C1—H1A	121.2	C13—C12—H12A	109.3
C2—C1—H1A	121.2	N2—C12—H12B	109.3
C3—C2—C1	120.51 (15)	C13—C12—H12B	109.3
C3—C2—H2A	119.7	H12A—C12—H12B	108.0
C1—C2—H2A	119.7	N3—C13—C12	110.78 (14)
F1—C3—C2	118.80 (16)	N3—C13—H13A	109.5
F1—C3—C4	118.20 (16)	C12—C13—H13A	109.5
C2—C3—C4	123.00 (16)	N3—C13—H13B	109.5
C3—C4—C5	116.40 (15)	C12—C13—H13B	109.5
C3—C4—H4A	121.8	H13A—C13—H13B	108.1
C5—C4—H4A	121.8	N3—C14—C15	111.70 (14)
C4—C5—C8	121.70 (14)	N3—C14—H14A	109.3
C4—C5—C6	130.89 (14)	C15—C14—H14A	109.3
C8—C5—C6	107.41 (13)	N3—C14—H14B	109.3
O1—C6—C5	130.57 (15)	C15—C14—H14B	109.3
O1—C6—C7	124.27 (15)	H14A—C14—H14B	107.9
C5—C6—C7	105.15 (12)	N2—C15—C14	111.42 (13)
O2—C7—N1	126.86 (15)	N2—C15—H15A	109.3
O2—C7—C6	127.14 (15)	C14—C15—H15A	109.3
N1—C7—C6	106.00 (13)	N2—C15—H15B	109.3
C1—C8—C5	120.76 (14)	C14—C15—H15B	109.3
C1—C8—N1	128.76 (14)	H15A—C15—H15B	108.0
C5—C8—N1	110.48 (13)	N3—C16—H16A	109.5
N1—C9—C10	111.95 (14)	N3—C16—H16B	109.5
N1—C9—H9A	109.2	H16A—C16—H16B	109.5
C10—C9—H9A	109.2	N3—C16—H16C	109.5
N1—C9—H9B	109.2	H16A—C16—H16C	109.5
C10—C9—H9B	109.2	H16B—C16—H16C	109.5
H9A—C9—H9B	107.9		
			
C8—C1—C2—C3	0.2 (3)	C7—N1—C8—C1	178.77 (17)
C1—C2—C3—F1	179.83 (18)	C9—N1—C8—C1	−6.3 (3)
C1—C2—C3—C4	0.3 (3)	C7—N1—C8—C5	−0.9 (2)
F1—C3—C4—C5	−179.72 (18)	C9—N1—C8—C5	174.01 (15)
C2—C3—C4—C5	−0.1 (3)	C7—N1—C9—C10	−80.2 (2)
C3—C4—C5—C8	−0.4 (3)	C8—N1—C9—C10	105.47 (19)
C3—C4—C5—C6	−179.75 (19)	N1—C9—C10—S2	175.74 (11)
C4—C5—C6—O1	1.0 (4)	C11—S2—C10—C9	87.13 (13)
C8—C5—C6—O1	−178.4 (2)	C12—N2—C11—S1	−168.35 (14)
C4—C5—C6—C7	−179.90 (19)	C15—N2—C11—S1	−6.2 (2)
C8—C5—C6—C7	0.70 (19)	C12—N2—C11—S2	11.7 (2)
C8—N1—C7—O2	−179.46 (18)	C15—N2—C11—S2	173.86 (12)
C9—N1—C7—O2	5.4 (3)	C10—S2—C11—N2	178.69 (12)
C8—N1—C7—C6	1.28 (18)	C10—S2—C11—S1	−1.28 (13)
C9—N1—C7—C6	−173.87 (15)	C11—N2—C12—C13	−150.62 (16)
O1—C6—C7—O2	−1.3 (3)	C15—N2—C12—C13	46.3 (2)
C5—C6—C7—O2	179.54 (18)	C14—N3—C13—C12	63.3 (2)
O1—C6—C7—N1	177.94 (19)	C16—N3—C13—C12	−175.16 (16)
C5—C6—C7—N1	−1.21 (18)	N2—C12—C13—N3	−55.4 (2)
C2—C1—C8—C5	−0.7 (3)	C13—N3—C14—C15	−62.61 (18)
C2—C1—C8—N1	179.63 (17)	C16—N3—C14—C15	175.73 (14)
C4—C5—C8—C1	0.9 (3)	C11—N2—C15—C14	151.30 (15)
C6—C5—C8—C1	−179.65 (16)	C12—N2—C15—C14	−45.1 (2)
C4—C5—C8—N1	−179.43 (16)	N3—C14—C15—N2	53.36 (19)
C6—C5—C8—N1	0.04 (19)		

### Hydrogen-bond geometry (Å, °)

**Table:**

*D*—H···*A*	*D*—H	H···*A*	*D*···*A*	*D*—H···*A*
C13—H13B···O2^i^	0.97	2.50	3.225 (2)	131
C12—H12A···O2^ii^	0.97	2.61	3.385 (2)	137
C15—H15B···O2^ii^	0.97	2.62	3.383 (2)	136
C1—H1A···O1^iii^	0.93	2.70	3.282 (3)	121
C2—H2A···O1^iii^	0.93	2.67	3.275 (2)	124
C12—H12B···S2^i^	0.97	2.97	3.866 (3)	155
C11—S1···Cg1^iv^	1.6673 (15)	3.403 (2)	3.695 (3)	86.43 (6)

Symmetry codes: (i) −*x*+1, −*y*+1, −*z*+1; (ii) −*x*+3/2, *y*+1/2, −*z*+3/2; (iii) −*x*+5/2, *y*+1/2, −*z*+3/2; (iv) −*x*+5/2, *y*+3/2, −*z*+3/2.

### Table 2 C═S···π-electron ring interactions (Å)

**Table:**

C═S···Cg	C···Cg	S···Cg
C11═S1···Cg1^i^	3.695 (3)	3.403 (2)

Symmetry code: (i) -x+2,-y+1,-z+1. Cg1 is the centroid of N1-C8-C5-C6-C7 (pyrrole).
